# ADAPTed Cognitive Behavioral Therapy for Pediatric Functional Abdominal Pain in Community-Based Pediatric Care: Mixed Methods Study

**DOI:** 10.2196/67106

**Published:** 2025-08-20

**Authors:** Emma Ramsay Milford, Sandra Buratti, Natoshia R Cunningham, Åsa Nilses, Ewa-Lena Bratt, Sandra Weineland

**Affiliations:** 1 Department of Psychology, University of Gothenburg, Haraldsgatan 1, Gothenburg, 413 14, Sweden, 46 0708276061; 2 Region Västra Götaland, Pediatric Healthcare, Regionhälsan, Gothenburg, Sweden; 3 Department of Family Medicine, Michigan State University, College of Human Medicine, Grand Rapids, MI, United States; 4 Region Västra Götaland, Center for Progress in Children's Mental Health, Regionhälsan, Gothenburg, Sweden; 5 Sahlgrenska Academy, University of Gothenburg, Gothenburg, Sweden; 6 Region Västra Götaland, Sahlgrenska University Hospital, Children's Heart Centre, Gothenburg, Sweden; 7 Region Västra Götaland, Primary Healthcare, Närhälsan, Södra Älvsborg, Sweden; 8 Sahlgrenska Academy, Institute of Medicine, General Practice/Family Medicine, School of Public Health/Community Medicine, Gothenburg, Sweden

**Keywords:** pediatric functional abdominal pain, digital intervention, cognitive behavioral therapy, primary care, cultural adaptation, psychological intervention, pediatrics, children, youth, mixed methods, patient experience, community-based, abdominal pain, anxiety, gastroenterology, child interview

## Abstract

**Background:**

The Aim to Decrease Anxiety and Pain Treatment (ADAPT) is a blended, digital, and live cognitive behavioral therapy program for children with functional abdominal pain disorder (FAPDs) and anxiety. Initially developed and evaluated in US pediatric gastroenterology settings, a culturally refined version was developed in Swedish, for potential use within community-based pediatric health care settings.

**Objective:**

This study aimed to evaluate a modified version of ADAPT as an early intervention for FAPD within a community-based pediatric setting in Sweden, exploring both the potential treatment effect and participants’ treatment experience.

**Methods:**

Participants were aged 9‐14 years, and all were diagnosed with FAPD. Using a mixed methods design, the study examined the preliminary effect through a single-arm pre-posttest and treatment experience through semistructured child interviews. Data were analyzed in three steps: nonparametric quantitative analysis of results on pre- and postintervention measures of self-rated pain-related functional disability, pain intensity, and anxiety; thematic qualitative analysis of the interviews; and conversion of qualitative data to enable both datasets to be analyzed and presented together.

**Results:**

A total of 13 children (12 girls) participated in ADAPT, all completing the program. In total, 7 of the invited 13 participating children agreed to be interviewed following intervention completion. Quantitative results were analyzed using the Wilcoxon signed rank test, and Pearson *r* was used to calculate the effect size. Results showed a significant reduction in pain-related functional disability, with a median decrease from 14.00 (IQR 10‐20) preintervention, to 5.00 (IQR 1‐9) postintervention (*P*=.04), a large effect size, *r*=−0.58, and 46% (n=6) achieving a clinically meaningful change where a Functional Disability Inventory (FDI) score decrease of ≥7.8 points denoted a clinically meaningful treatment response. Pain intensity also significantly decreased from a median of 6.5 (IQR 5.25‐8) to 4.00 (IQR 2.5‐6.5; *P*=.02), a large effect size *r*=−0.70, with 33% (n=4) experiencing at least a 50% reduction. Clinically meaningful change was determined to be present if at least a 50% reduction in self-rated measures of pain intensity was observed. Qualitative thematic analysis identified three themes: “Starting from scratch,” “Experiencing the treatment,” and “Getting on with life.” In terms of treatment experience, the blended live or digital format was perceived as a good fit for youth. Most children described finding some strategy that was effective and reported positive outcomes, such as increased participation in school.

**Conclusions:**

As patient experiences were predominantly positive and quantitative results indicative of potential for increased function and reduced pain, our findings suggest that ADAPT may present as a possible early treatment option for FAPD in a Swedish community–based pediatric setting. To draw robust conclusions on effectiveness, further research is required using a larger sample size, as well as research aimed at following up treatment effects over time.

## Introduction

Functional abdominal pain disorders (FAPD) are gastrointestinal (GI) symptoms where medical evaluation does not reveal any underlying organic cause. FAPD is diagnosed following the Rome IV criteria, where symptom frequency and duration are taken into account when giving a diagnosis. The location of the abdominal pain, along with the type of gastro or intestinal symptoms, can enable the physician to assign a specific FAPD subtype diagnosis, such as irritable bowel syndrome (IBS), associated with changes in bowel patterns, such as constipation and diarrhea, or functional dyspepsia when upper epigastric pain is present. When criteria for a specific subtype are not met, a more general diagnosis, such as functional abdominal pain not otherwise specified, is given. Pediatric FAPD is a GI condition affecting up to 25% of children [1,2]. FAPD is associated with significant impairment, including academic impairment and poor quality of life, increased risk of developing other pain conditions, and long-term vulnerability to anxiety even if pain resolves [3-8]. Mental health comorbidities, such as anxiety, are common and associated with adverse pain-related outcomes [9-12]. FAPD also poses a considerable economic burden on patients, families, and health care systems [13,14]. Hence, developing and evaluating effective and early treatments is of great importance.

Although there is insufficient evidence supporting pharmacological treatments [15], including antidepressants [16] or probiotics [17], psychosocial interventions, such as cognitive behavioral therapy (CBT), improve pain levels [18], functional disability [19], and quality of life [20,21]. Internet-delivered exposure-based CBT has been found to be both an effective and cost-effective treatment for children with FAPD [21]. Anxiety impacts response to CBT for pain in youth [10]. Thus, Aim to Decrease Anxiety and Pain Treatment (ADAPT) was developed to address both pain and anxiety in youth with FAPD [22]. It was initially developed from evidence-based CBT protocols for the management of pediatric pain [23] and childhood anxiety disorders [24].

Psychological interventions such as CBT for pediatric patients presenting with pain [25] and FAPD [19] have been shown to be effective and of particular importance, as research has shown a connection between FAPD and anxiety [26]. Although comorbid anxiety is common among children with FAPD, it is more usual that gut symptoms occur first and that psychological distress is secondary [27]. Psychological interventions for children with FAPD can be effective in increasing the ability to participate and function in daily activities and alleviating symptoms of anxiety [19,21]. Pain-related functional disability refers to limitations in everyday life activities. Children with pain conditions such as FAPD are commonly limited in their ability to attend school and engage in physical and social activities [28]. Functional disability can co-occur with anxiety, which can adversely impact children’s response to CBT [10].

Previous research conducted in Sweden, aimed at evaluating internet-delivered, exposure-based psychological treatment for IBS, a specific subtype of FAPD, has indicated both effective results and cost-effectiveness [20,21]. Exposure-based CBT typically focuses on avoidance behavior and, therefore, is suited to patients who, due to their GI symptoms, avoid pain- and anxiety-inducing stimuli. Exposure therapy aims to decrease fear of symptoms and reduce avoidant behaviors by gradual exposure to symptoms and feared situations and aversive stimuli. The mediating role of exposure has been evaluated in adolescents with IBS [29]. Change in GI-specific anxiety and GI-specific avoidance has been found to mediate changes in parent-reported abdominal symptoms for children with FAPD receiving exposure-based internet-CBT. Children with high baseline values of GI-anxiety and GI-avoidance are likely to benefit more from exposure-based internet CBT than children with low symptom-specific fear and avoidance [30]. Although common in patients with FAPD, not all experience symptom-related fear or display avoidance behavior, particularly those with less severe symptoms. Avoidance behaviors and ineffective pain-coping strategies may be learned from parents [31]. Hence, learning CBT strategies to manage pain in combination with anxiety management strategies at early onset FAPD could be a valuable contribution and treatment option within community-based care. Although the long-term effectiveness of broader multicomponent CBT, such as ADAPT, where several strategies are included as opposed to exposure-focused therapy, has been questioned [29]. However, it can be argued that there is a need for a range of CBT programs in order to provide treatment to children with varying FAPD symptoms. Furthermore, there is a need to develop and implement more treatment programs that can be scaled up and delivered using web-based approaches, both due to the large number of children and young people with FAPD and to keep in line with current developments to make health care more equitable and accessible.

The theoretical underpinnings of ADAPT are grounded in CBT and combine classic components such as cognitive restructuring and exposure with aspects of third-wave CBT [32]. This wave of CBT challenges the notion of behavioral and cognitive change as the only active components and places emphasis on the patient’s perception and acceptance of their situation. The struggle to avoid unpleasant and undesirable events, thoughts, and feelings is central to CBT, which has its roots in learning theory. Third-wave CBT focuses on psychological flexibility, requiring acceptance of the concept that some things are not within our control. The ability to be mindful, in the moment, and focus inwards is an aspect of third-wave CBT that is incorporated into ADAPT and the strategies taught through the program. The ADAPT protocol is a CBT protocol and should not be confused with acceptance and commitment therapy. Acceptance and commitment therapy as a pain treatment aims to increase successful engagement in activities that are meaningful and promote vitality for those who live with persistent pain. The classic CBT components of the ADAPT program are developed from the evidence-based CBT protocol “Cool Kids” for childhood anxiety disorders [24]. ADAPT is also grounded in the gate control theory of pain, which asserts that pain can be inhibited by activation of nerves that do not transmit pain signals. The pain management components of the ADAPT program are developed from an evidence-based protocol for pediatric pain [23]. ADAPT is associated with improvements in disability and anxiety levels as compared to standard medical care in youth with FAPD presenting to pediatric gastroenterology clinics [19].

ADAPT was redeveloped into a Swedish language version by two Swedish clinical psychologists (ÅN and ERM) and the original author of the American version (NRC). Portions of program content (live videos within web modules) were redesigned as animated films. Additionally, cultural adjustments to the original protocol were made (see Multimedia Appendix 1 for an overview of treatment content and session breakdown and Multimedia Appendix 2 for an overview of the original treatment and adapted Swedish language version). The cultural adaptation involved altering both content and language to align with a Swedish cultural setting. This involved changes such as altering terminology that is culturally specific to the United States, for example, spring break, and adjusting references to school grade terminology to align with the Swedish system. The module “Building Social Skills: How to be assertive” in the original version was left out of the Swedish version, as in Swedish school and society, children are not expected to handle bullying incidents. It is stipulated in Swedish educational law that children should not be subject to bullying in school; therefore, the responsibility is placed solely on adults to prevent bullying, and it would not have been appropriate to include this module in the treatment program. The adaptation involved going from videos with actors to creating animated characters, which also allowed for the opportunity to create an inclusive and culturally appropriate visual profile. The translation of the manual and manuscripts was done in several steps. Initially, an authorized translator was used. Following this, the texts were perused by clinical psychologists to ensure appropriate medical and psychological terminology was used. Finding appropriate Swedish names for ADAPT strategies was also part of the adaptation process. Some names were not possible to translate directly due to language differences—for example, calming statements, activity pacing, and pleasant activities, in which case, new terminology had to be applied. The cultural adaptation also involved finding appropriate names for the characters in the Swedish version.

An important reason to evaluate psychological treatment within a community-based pediatric setting is that it is in line with the ambition to decrease utilization of acute health care and offer earlier treatment. The importance of moving from reaction to prevention in relation to FAPD has been highlighted [33]. Multidisciplinary care for children with persistent FAPD has been associated with reduced utilization of acute health care [34]. It has been suggested that a multidisciplinary approach be used for optimal care of children with FAPD, but it is also implied that this may not always be feasible in primary care settings [35]. There is a need for accessible resources to enable FAPD to be managed within first-line health care, as well as practical strategies to assist primary care providers in the recognition and prompt diagnosis of FAPDs for early treatment interventions. It is therefore of importance to evaluate targeted treatments such as ADAPT that can be implemented as an early intervention.

A community-based pediatric setting that incorporates integrated medical assessment, diagnosis, and psychological treatment, such as ADAPT, could provide multidisciplinary care that may have a positive impact on reducing acute health care utilization for children with persistent symptoms. It could be that an integrated approach, including ADAPT as a psychological intervention for pain management within community-based pediatric care, could act as a preventative measure, thereby reducing referrals for repeated assessment and delaying treatment. Although excessive testing should be avoided, it is not uncommon that children with FAPD are subjected to numerous invasive and noninvasive tests. Extensive testing has not been found to have any impact on outcome or patient satisfaction [36]. The impact of pediatric FAPD on the child, parents, and society is substantial. The annual cost of health care for pediatric FAPD was explored in a Dutch study [13] where the cost per patient was estimated at approximately 2500 euros (US $2775). Inpatient and outpatient health care use were major cost drivers, followed by parental productivity loss. Differences in national health care structures prevent these figures from being broadly applied; however, the economic burden of pediatric FAPD can be concluded as being significant, which further implies the urgency of early access to treatment.

This formative study aims to evaluate a modified version of ADAPT as an early intervention for FAPD within a community-based pediatric care setting in Sweden. This is of importance as there is no prior research that has evaluated the potential of a CBT treatment program where all participants were recruited at this level of health care. Furthermore, this study is novel in that it is conducted within a clinical setting where recruitment was integrated into regular care. Due to the limited access to psychological treatment for pediatric FAPD, it is of importance to culturally adapt treatments available and evaluated in other countries, contexts, and languages. In addition to evaluating the effectiveness and accessibility of an adapted treatment program, it is equally important to evaluate children’s experiences of the treatment. There is a shortage of research that has aimed to explore children’s experiences of internet-delivered CBT for FAPD, and the need to access the opinions of children has been highlighted [37]. With formative research conducted in clinical settings, it is particularly important to obtain both quantitative and qualitative data. This mixed methodology contributes to an enhanced understanding of both the potential treatment effect and the participant’s treatment experience. Both aspects are invaluable when making decisions regarding implementing a treatment into clinical practice. Previous research focused on participants’ perceptions of ADAPT in relation to feasibility, tolerability, and patient outcomes has yielded valuable information in terms of understanding the potential of the treatment [22]. This study aims to explore experiential factors and outcomes further through an analysis of the congruence between children’s reported experiences of ADAPT and their results on pain-related outcome measures.

## Methods

### Design

A mixed methods approach [38] was used with a quantitative single-arm pre- and posttest design to explore treatment effect, a qualitative design with semistructured child interviews to explore experience of the treatment, and a conversion of qualitative data to enable both datasets to be analyzed and presented together. The study was approved by the Swedish Ethics Authority (Dnr 2022-03835-02).

### Measures

The Functional Disability Inventory (FDI) was used in order to evaluate the level of self-rated pain-related functional disability[39]. The FDI is a 15-item measure of limitations in children’s physical and psychosocial functioning due to their physical health. The psychometric properties of the FDI have been evaluated, confirming the reliability and validity of the measure for functional assessment of pediatric patients with chronic pain. Validity was found to be supported by significant correlations of child- and parent-report FDI scores with measures of school-related disability, pain, and somatic symptoms [39]. An example item is: “Being in school all day.” The answers are given on a scale ranging from 0 to 4, where 0=No trouble, 1=A little trouble, 2=Some trouble, 3=A lot of trouble, and 4=Impossible. An FDI score decrease of ≥7.8 points was used as an indicator of clinically meaningful treatment response in a previous evaluation of ADAPT [19], based on previous research on pediatric pain [40]. Hence, the same indicator was applied in this study.

Self-rated level of anxiety was measured using the Screen for Child Anxiety Related Disorders (SCARED) [41]. The SCARED is a 41-item measure of anxiety over 3 months, regularly used in pediatric pain research. The psychometric properties of SCARED have been evaluated, indicating robust properties and that the measure is both clinically relevant and can be used in research to monitor intervention effectiveness [42]. Higher scores indicate greater anxiety. An example item is “I worry about being as good as other kids.” The response items range from 0 to 2, where 0=Not true or Hardly ever true, 1=Somewhat true or Sometimes true, and 2=Very true or Often true. Clinical (**≥**25) and subclinical cut-offs (≥12) have been developed for children with chronic pain [43]. Given the adaptation of this program into pediatric community-based care, it was deemed that the subclinical range of anxiety would be optimal to identify patients who may benefit from an early intervention. Hence, patients with a self-rated score of ≥12 were eligible to participate.

Pain intensity was measured using a visual analogue scale (VAS) of 1‐10 with both digits and faces. The psychometric properties of VAS for children with chronic abdominal pain have been evaluated, indicating strong correlations with other measures aimed at measuring pain [44].

The interview guide was semistructured, based on the semistructured interview used in the original study [22] and included questions about experience, and the impact of ADAPT (see Multimedia Appendix 3 for interview protocol).

### Participants

Participating patients were aged 9‐14 years and diagnosed with FAPD at a community-based pediatric health clinic. The criteria for inclusion were the presence of child-reported subclinical anxiety (total score ≥12), as measured using the SCARED [41,45] or pain-related functional disability as measured by the FDI [39,46], total score ≥7 for study participation. Participants had no organic painful medical conditions nor any identified learning disabilities. Participants were all Swedish language speakers. All participants were assessed by a licensed pediatric psychologist.

### Procedure

Participants in this study were recruited from a Swedish community-based pediatric health clinic, which is a specialist clinic within regional care, organizationally located between primary care and hospital-based specialist services. While not traditional primary care, these clinics are part of the public health care system, do not require a hospital referral, and are often the first point of contact for pediatric patients with chronic and complex health care issues*.* Recruitment took place within the clinical setting. Participating children were referred to the clinic by their general practitioner. After FAPD was confirmed by a pediatrician at the clinic, children were referred, in-house, to a multidisciplinary team at the clinic. The children were then screened for pain-related functional disability with the FDI and presence of worry using a digital patient questionnaire as part of regular clinical practice (refer to Multimedia Appendix 4 for screening questionnaire). Children with an indication of functional disability and a yes response to a question regarding the presence of worry or stress were then assessed for inclusion by a pediatric psychologist at the clinic. Inclusion was based on participants’ age, clinical judgment, and assessment, where children with scores below 12 on the SCARED or no presence of pain-related functional disability on the FDI were not deemed to need individual intervention and therefore excluded from the study. Excluded patients were allocated to another intervention where their parents were offered the opportunity to participate in a 2-hour group-based educational training held by a pediatric psychologist and a pediatric nurse. Patients with an FDI score of ≥7 or the presence of worry and avoidance behavior were approached to participate in the study. A psychologist informed the patients, children, and their carers, both verbally and in writing, about the study, whereby written consent was obtained from the parents of 13 participants, all of whom completed the intervention and quantitative assessment measures. All participating children consented verbally. Participants were approached by a psychologist during the final intervention session and asked whether they would consider participating in an optional qualitative interview about their experience of the program. In total, 7 participants (1 assigned male at birth and 6 assigned female at birth) were interviewed approximately 1 month postcompletion of ADAPT.

Data were collected between January 2022 and December 2023, and recruitment was consecutive during the initial 18 months of the period.

### Intervention

ADAPT [22] is a blended, digital, or face-to-face CBT protocol targeted toward pediatric FAPD and anxiety symptoms. ADAPT is brief, consisting of two clinic-based sessions and four self-paced web sessions. Each self-paced web module was followed by a digital session (video call) with a psychologist. The treatment sessions were held at weekly intervals, allowing for a treatment period of approximately 6 weeks. ADAPT was delivered by three clinical psychologists affiliated with the participating clinic. Parents participated in both the clinic-based and digital sessions. In line with the treatment manual [22], the pain-coping strategies (deep-breathing, mindful breathing, guided imagery, progressive muscle relaxation, calming statements, and activity pacing) were taught to the child alone. The instructions were then repeated to the child and parent to facilitate practice between sessions. The duration of the two clinic sessions was approximately 1 hour, and the digital sessions with the clinical psychologist were approximately 20 minutes.

### Analyses

The data were analyzed in three steps: (1) quantitative analysis of results on pre- and postintervention measures of self-rated pain-related functional disability, pain intensity, and anxiety; (2) qualitative analysis of the interviews; and (3) integration of results through assessment of the congruence of quantitative and qualitative results.

#### Step 1: Quantitative Analysis

Descriptive information and changes in outcomes at the group level, functional disability, pain intensity, and anxiety were explored via nonparametric Wilcoxon signed rank tests using SPSS (version 29; IBM Corp) software. Pearson *r* [47] was used to calculate effect size, where results are referred to as small (−0.1 to −0.3), medium (−0.3 to −0.5), or large (−0.5 or less). Reference points for clinically meaningful change in functional disability from previous evaluations of ADAPT were used. Hence, a total FDI score of 3.5 was considered indicative of healthy youth, and an FDI score decrease of ≥7.8 points was considered indicative of a clinically meaningful treatment response [19]. Clinically meaningful change was determined to be present if at least a 50% reduction in self-rated measures of pain intensity was observed [48]. A 50% reduction in scores on SCARED at posttreatment was also considered a clinically meaningful indicator of improvement or remission. Reduction of 50% on the SCARED has been found to optimally predict treatment response and can be used to systematically monitor treatment outcomes [49].

All participants completed the treatment and self-rated postmeasures. There was no missing data among postintervention measures; however, one participant’s preintervention measure of pain intensity was missing due to an administrative error. Hence, n=12 for VAS preintervention.

#### Step 2: Qualitative Analysis

Reflexive thematic analysis was carried out by two researchers (ERM and ÅN) as part of the research project. Data were approached from a critical realist position as the participants’ subjective descriptions of their experiences were obtained, and the researchers’ roles and subjectivity were also recognized as informing the research. A critical realistic position in the context of reflexive thematic analysis takes the view that participants’ perceptions are shaped by their own contexts and representations of reality, which are then interpreted and analyzed, and thereby shaped by the experiential and cultural context held by the researcher. The aim of thematic analysis from a critical realist standpoint is to establish a lucid and intelligible interpretation of the participants’ experiences that resounds with situational realities [50].

To ensure comprehensive analysis, a team was assembled, consisting of three coauthors: ERM, a licensed psychologist and doctoral student; ÅN, a licensed psychologist; and SW, an associate professor, supervisor, and licensed psychologist. Drawing from our collective understanding and experience of internet-delivered CBT as an effective treatment for adults, we initiated the analysis process. Through iterative discussions, themes, subthemes, and categories were collaboratively refined and defined until consensus was achieved among the analysis team, initially comprising authors ERM and ÅN, and later including SW, SB, and ELB. Notably, ERM and ÅN’s analysis was overseen by SW, progressing from themes and subthemes to categories, initial codes, and ultimately quotes, ensuring the reliability of the findings. Atlas.ti (version 23; ATLAS.ti Scientific Software Development GmbH) software was used as support in data processing.

#### Step 3: Convergent Analysis

A conversion of qualitative data was performed to enable both datasets to be analyzed together and presented in a joint display table [38]. During this process, themes were analyzed again, comparing them to individual responses. Responses were then reduced into a congruence measure to indicate to which extent the individual’s responses aligned with the theme. Although the themes were drawn from the entire dataset and thereby guided by the individual responses, there was a range of responses where some were more congruent with or aligned to the theme. This analysis enabled any patterns between self-rated measures and the level of congruence with subthemes to be observed. A low congruence score was applied when the individual responses generally aligned with the subtheme, but some responses were conflicting. A medium congruence score was applied when the individual’s responses generally aligned with the subtheme, but some responses were neutral. A high congruence score was applied when all the individual’s responses aligned with the theme.

### Ethical Considerations

The study adhered to the ethical principles outlined in the World Medical Association’s Declaration of Helsinki. Participation was entirely voluntary. Participants and their guardians were provided with written and verbal information about the study. Guardians provided written informed consent, and participants provided verbal assent. The study received ethical approval from the Swedish Ethics Review Authority (2022-03835-02). All participants and their legal guardians were informed about the study during an initial psychological assessment session. Guardians and patients subsequently attended an appointment for feedback regarding the assessment. If the participant met the inclusion criteria, participation in the study was offered. Upon agreement, the guardian signed a written consent form, and the participant provided verbal assent. This process also applied to secondary data used in this analysis; the original consent included approval for secondary analysis. To ensure participant confidentiality, all collected data were deidentified and assigned numerical codes. No personally identifiable information was retained in the analytic dataset. Audio recordings were securely stored. Access to the data was restricted to the research team only. Data handling and storage complied with the General Data Protection Regulation and the data management policies of Regionhälsan (Region Västra Götaland), including secure storage on encrypted devices and restricted physical access in accordance with institutional guidelines.

## Results

### Quantified Change

**Figure 1.**
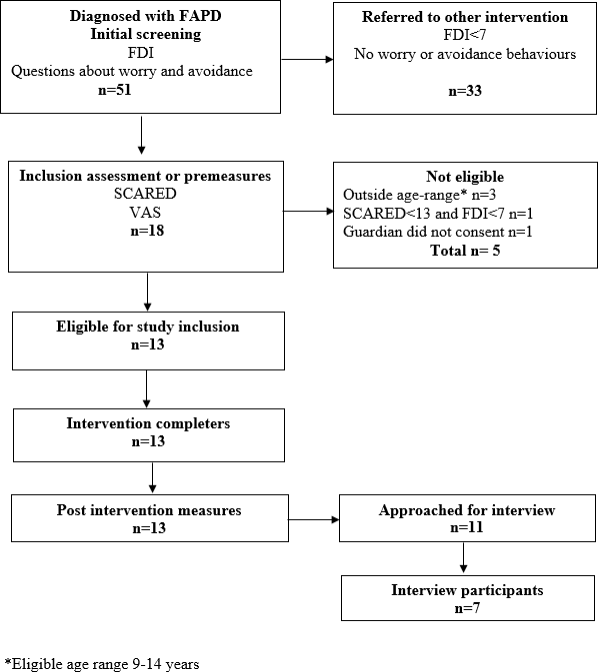
Allocation process and included participants. FAPD: functional abdominal pain disorders; FDI: Functional Disability Inventory; SCARED: Screen for Child Anxiety Related Disorders.

Results on measures of pain-related functional disability indicate a statistically significant decrease in disability from preintervention, median 14.00 (IQR 10‐20), indicative of moderate disability; range 10‐20, to postintervention, median 5.00 (IQR 1‐9), indicative of mild disability; n=13, and a large effect size, *z* score=−2.10; *P*=.04; *r*=−0.58. Given the 12-point change in disability, 46% (n=6) achieved a clinically significant [40] change (>7.8 points). Approximately 85% (11/13) of participants experienced decreased levels of pain-related functional disability (median change=8). The inclusion process and measurement points are provided in Figure 1.

Measures of pain intensity were also indicative of a statistically significant decrease from preintervention, median 6.5 (IQR 5.25‐8), n=12, to postintervention, median 4.00 (IQR 2.5‐6.5), n=13, and a large effect size was found, *z* score=−2.42; *P*=.02; *r*=−0.70. Clinically meaningful [48] pain reduction was observed in 4 of 12 (33%) participants who experienced at least a 50% reduction in pain intensity scores after ADAPT. Approximately 83% (10/12) of participants experienced decreased levels of pain intensity.

A total of 6 (46%) participants experienced at least some reduction across all three outcomes, with the 7 remaining participants experiencing a reduction in one (8%) or two (38%) categories. One participant did not improve in any category.

### Qualitative Insights—Treatment Experience

Thematic analysis of the seven interviews resulted in three main themes and seven subthemes. The themes and subthemes are presented in Textbox 1 (see Multimedia Appendix 5 for themes, subthemes, and corresponding quotes from child interviews).

Textbox 1. **Themes and subthemes**

**Starting from scratch**

- No experience of psychological treatment
- Uncertain expectations

**Experiencing the treatment**

- Getting on with the content
- Engaging with a therapist
- Managing the digital format
- Getting help from a parent or carer

**Getting on with life**

- Using some of a range of strategies
- Learning about pain and worry
- Doing things despite symptoms

### Starting From Scratch

This theme draws upon the children’s expressions about thoughts, knowledge, and expectations of the treatment prior to commencing. Two subthemes were found, one relating to experience and the other to expectations. The overarching theme draws on the finding that children expressed both having little experience and being uncertain about what to expect from it.

### Experiencing the Treatment

This theme relates to the children’s attitude toward and experience of the blended format of the treatment, with the in-person sessions with a therapist, video calls, and digital modules including the animated films, as well as how they perceived the content. The digital content, such as films and assignments, was found to be acceptable by most participants. The content was perceived by some participants as somewhat tedious, and characters and examples were not found to be relatable by all participants. However, not being able to relate to the characters was not described as problematic. Generally, the blended format was perceived positively. Children made positive comments about the interaction with the therapist, but also mentioned the benefits of working from home. Saving time and choosing when to do the work was mentioned. Meeting the therapist was mostly appreciated and by some referred to as “real.” Most of the participants expressed getting and needing help from a parent.

### Getting on With Life

This theme captures the children’s experiences of the strategies and perceived outcomes of the treatment. The children commented on the strategies, indicating a preference for some over others. Pain coping strategies were mentioned more than the strategies targeting anxiety. Most of the children gave examples of having applied some strategy. Some children reported increased understanding of symptoms of pain and worry. Increased participation in school was mentioned by most children as being the main impact of the program on their functioning.

### Mixed Methods Integrated Results

Participant responses obtained through interviews varied in their level of congruence with established subthemes. The integrated results for the seven interviewed participants are displayed in Table 1. Most of the participants made comments that had a high to medium level of congruence with subthemes, but some responses were less or not congruent. The mixed methods joint display enables observation of patterns in responses and self-rated change. The convergent results in Table 1 suggest that patients with a low level of congruence on two or more subthemes are those whose ratings on postintervention measures increased. In general, a match between a high level of congruence and an overall decrease between pre- and postintervention ratings was observed.

**Table 1.** Pre- and postintervention results on self-rated measures of pain-related functional disability, anxiety, pain intensity, subthemes, and level of congruence between responses and subthemes among interviewed participants (n=7)^a^.

	Participants^b^ (age in years)						
	Ellen (10)	Alex (11)	Molly (9)	Julie (14)	Sofia (12)	Anne (12)	Lara (12)
Measures							
Functional Disability Inventory preintervention: median 16 (IQR 12.5-20; 14)	24	16	9	29	11	14	16
Functional Disability Inventory postintervention: median 6 (IQR 4.5-8.5; 5)	0	4	16	6	5	7	10
Change	−24	−12	+7	−23	−6	−7	−6
Pain intensity rating preintervention 0‐10: median 7 (IQR 6.0-7.5; 6.5)	8	7	8	6	6	7	4
Pain intensity rating postintervention 0‐10: median 5 (IQR 2.5-6.5; 4)	7	5	10	2	2	6	3
Change	−1	−2	+2	−4	−4	−1	−1
Screen for Child Anxiety Related Disorders preintervention: median 23 (IQR 16.0-33.5; 21)	12	13	39	37	30	23	19
Screen for Child Anxiety Related Disorders postintervention: median 23 (IQR 12.0-30.0; 23)	7	23	54	34	14	26	10
Change	−5	+10	+15	−3	16	+3	−9
Subthemes (congruence between responses and subthemes^c^)							
No experience of psychological treatment	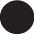	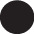	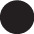	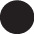	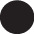	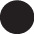	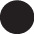
Uncertain expectations	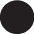	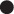	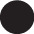	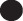	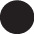	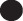	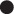
Getting on with the content	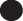	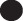	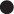	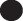	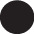	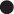	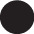
Getting help from a parent or carer	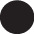	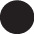	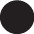	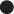	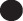	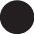	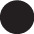
Engaging with a therapist	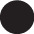	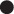	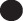	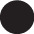	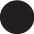	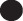	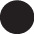
Managing the digital format	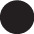	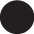	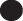	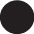	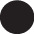	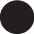	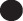
Using some of a range of strategies	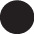	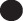	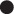	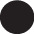	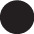	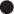	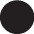
Learning about pain and worry	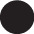	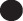	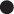	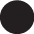	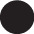	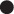	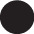
Doing things despite symptoms	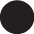	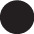	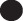	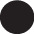	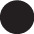	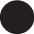	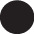

a Median scores for participants included in mixed methods analysis (n=7) are shown, as well as those for all intervention completers (n=13, shown in brackets) to enable comparison between scores for mixed methods participants and whole group.

b Not real names. Median age 12 (n=7).

c Interpretation key for level of congruence: low: 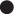; medium: 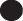; high: 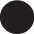.

## Discussion

### Principal Findings

Pediatric FAPD is common, associated with significant impairment, increased risk of developing other pain conditions, and long-term vulnerability to anxiety. Furthermore, the condition poses a considerable economic burden on patients, families, and health care systems. CBT as a treatment for FAPD has previously been found to be effective, and ADAPT has been evaluated for patients recruited from pediatric gastroenterology departments. This study evaluated a modified version of ADAPT as an early intervention for FAPD within a community-based pediatric care setting in Sweden.

Findings from this study indicate that ADAPT is a potentially effective and suitable treatment for pediatric FAPD within pediatric community-based clinics, particularly for pain-related outcomes. Qualitative results indicate that several interviewed patients reported positive experiences, and where the reported experience was mixed, positive quantitative outcomes were observed in at least one domain of outcome variables for most participants. The results are in line with previous studies [19,22], reinforcing the efficacy of ADAPT as a potentially viable therapeutic approach and broadening its usability to a community-based pediatric care context. Treating patients early when their symptoms are less compounded may prevent more long-term medical and associated mental health difficulties. Prior research into pediatric FAPD has generally focused on patients at secondary or tertiary level of care [19-22] and has not, as was done in this study, solely recruited patients seeking treatment within regular clinical care at pediatric community-based clinics. This may have represented a missed opportunity for earlier and more accessible care.

At the group level, our findings indicate both statistically and clinically significant changes in terms of pain-related functional disability and pain intensity. Due to the small sample size and single-arm design, these findings cannot be generalized and will need to be replicated in a controlled trial with more participants to draw conclusions about the treatment effect. Hence, next steps should include conducting larger, controlled studies to evaluate the treatment’s effectiveness more robustly, as well as exploring long-term outcomes and mechanisms of change. One limitation of this study relates to the use of cut-off scores on the SCARED instrument. The use of categorical cut-offs in an uncontrolled, observational context may be problematic. Cut-offs can impose artificial dichotomies on what is inherently a dimensional construct and may lead to a loss of information about subclinical variations in symptom severity. In the absence of a control group, reliance on such thresholds may also overestimate or underestimate clinically meaningful change. Future studies should consider supplementing cut-off-based classifications with dimensional analyses to better capture nuanced symptom trajectories over time*.*

There was no significant change in measures of anxiety. Although individuals with subclinical anxiety were recruited, the sample also contained participants with higher levels of anxiety, either approaching or within the clinical range. It may be that patients with subclinical levels of anxiety could benefit from early treatment, particularly in terms of impact on pain-related outcomes. This would need to be explored further in future research, ideally with a larger sample size, before concluding whether early interventions delivered through community-based pediatric care have the potential to prevent the onset of a formal anxiety disorder diagnosis.

Future research may wish to address participants’ quality of life and other mental health comorbidities to explore potential changes and issues that may impact the treatment effect. It would also be of interest to explore whether participants’ anxiety was pain-related, specific, or more general, and if these aspects affect outcomes. Furthermore, repeated measurements at follow-up intervals would enable a greater understanding of any changes in anxiety and treatment effect over time. As increased participation in school was mentioned by participants as an improvement to their functioning following the intervention, it would have been of interest to explore this in greater detail, particularly the presence of significant differences on specific questionnaire items relating to school attendance. This was not possible in this study due to the design and method of data collection. Future research may wish to explore ADAPT’s potential specifically with regard to school attendance by measuring change on specific items within the FDI and SCARED relating to school.

The blended format appears to be a good fit as participants expressed both advantages of engaging with the therapist, as well as the digital format. Children’s interview responses indicate that the web-based format allowed them to use their time more effectively; however, the personal connection obtained through clinic visits was also appreciated. There is evidence that any live intervention (whether in person or digital) is comparable [51]. Yet, future research may wish to explore whether face-to-face intervention in clinics differs from face-to-face intervention delivered via digital channels, such as video calls, specifically for this population. Such research could provide insight into whether components of blended treatments can be adapted further to increase treatment accessibility and patient satisfaction. All participants completed the whole treatment and all modules, and the reasons for the good compliance could be explored further.

Although psychological treatment programs are not expected to be equally effective for all patients, children’s views can contribute important information in terms of improving outcomes. Accessing children’s views is both relevant in terms of the United Nations conventions on the rights of the child and also in line with the current movement toward patient-centered care. Our findings indicate that children use at least one out of a range of strategies. These findings imply that there may be a demand for CBT for pediatric FAPD that includes a range of strategies. This would need to be explored further by obtaining children’s views on a range of CBT components in further depth.

A great deal of effort was placed on constructing a visual profile that was representative of genders, ethnicity, and ability, and providing a range of examples of FAPD and worry. This did not appear to have impacted some children’s views of how relatable the content was. However, as the findings indicate potential treatment effects on pain and functional disability, it may be that being able to relate to characters was considered more important by the treatment creators than by the participating children. This could be explored further in future studies seeking to evaluate factors contributing to the treatment effect. Furthermore, it is worth considering whether creating a treatment program with a team of adults and then asking children about their views of the content is the optimal design process. Perhaps cocreating the characters and using children’s own examples as content would have been a better option. This should be considered in future projects aimed at designing digital treatments aimed at children.

### Strengths and Limitations

A key strength of this study lies in its focus on the feasibility and acceptability of the ADAPT intervention within a real-world clinical context. By conducting the study in a community-based pediatric care setting, we were able to explore how the intervention functions under routine clinical conditions. The use of a mixed methods design further enhances the value of the findings, allowing for integration of both quantitative outcomes and qualitative data into participants’ experiences. This dual perspective provides a richer understanding of how the intervention was received by children and which components may contribute to meaningful change.

However, several limitations should be acknowledged. The small sample size limits the generalizability of the findings and reduces the statistical power to detect more subtle effects. This constraint is partly due to the study being conducted within a single clinical setting and under limited funding conditions. Despite these limitations, the study offers an important foundation for future development and scale-up. Overall, this study contributes valuable insights into how a structured psychological intervention can be implemented and received in community-based care and serves as a foundation for continued adaptation and integration.

Transitioning psychological treatment for FAPD from hospital-based care to pediatric clinics offers several important benefits. These clinics often represent the first point of contact for children with recurrent abdominal pain, making them an ideal setting for early identification and intervention. Providing access to evidence-based interventions such as CBT could improve treatment accessibility, reduce referral delays, and decrease the burden on hospitals. Early intervention may also prevent the development of chronic symptoms, reduce unnecessary medical investigations, and improve overall outcomes for both children and families. Embedding interventions like ADAPT in community-based pediatric care could be an important step toward more integrated, potentially preventive, and cost-effective management of pediatric FAPD.

### Conclusions

This study is the first of its kind to evaluate the use of ADAPT through regular clinical routine within a pediatric community-based care context. The findings may provide a way forward for early, potentially effective, and accessible treatment for FAPD, as well as shedding light on patients’ treatment experience. For robust conclusions to be drawn, future research would need to expand upon these findings, further exploring ADAPT’s potential as an accessible treatment for pediatric FAPD available within community-based care.

## Supplementary material

10.2196/67106 Multimedia Appendix 1 Adapt content and session breakdown.

10.2196/67106 Multimedia Appendix 2 Overview of the original treatment and Swedish language version.

10.2196/67106 Multimedia Appendix 3 Interview protocol.

10.2196/67106 Multimedia Appendix 4 Questions about abdominal pain and worry.

10.2196/67106 Multimedia Appendix 5 Themes, subthemes, and related quotes.

## Abbreviations

ADAPT: Aim to Decrease Anxiety and Pain Treatment
CBT: cognitive behavioral therapy
FAPD: functional abdominal pain disorder
FDI: Functional Disability Inventory
GI: gastrointestinal
IBS: irritable bowel syndrome
SCARED: Screen for Child Anxiety Related Disorders
VAS: visual analogue scale
