# Supplementary material for: ADAPTed Cognitive Behavioral Therapy for Pediatric Functional Abdominal Pain in Community-Based Pediatric Care: Mixed Methods Study
Source: JMIR Form Res. 2025 Aug 20;9:e67106. doi: 10.2196/67106 (PMC12367232; doi:10.2196/67106)
Supplement: Multimedia Appendix 2 [file formative-v9-e67106-s002.docx]

| **Original English language version** | | **Swedish language version** | |
| --- | --- | --- | --- |
| **Session 1**  In person/clinic  60 minutes | Program overview, psychoeducation, deep breathing, and guided imagery | **Session 1**  In person/clinic  60 minutes | Program overview, psychoeducation, deep breathing, and guided imagery |
| **Session 2**  60 minutes  In person/clinic | Progressive muscle relaxation, calming statements, and activity pacing | **Session 2**  60 minutes  In person/clinic | Progressive muscle relaxation, calming statements, and activity pacing |
|  |  | **Videocall**  **15 minutes** | Preparing for digital modules. Run through of digital content session 3 |
| **Session 3**  45 minutes  Web/phone  (Child focus with parent support) | Pleasant activities and how to solve problems | **Session 3**  45 minutes  Videocall  Child and parent | Pleasant activities and how to solve problems |
| **Session 4**  45 minutes  Web/phone  (Child focus with parent support) | Detective Thinking | **Session 4**  45 minutes  Videocall  Child and parent | Detective Thinking |
| **Session 5**  45 minutes  Web/phone  (Child focus with parent support) | Fighting fears by facing fears & How to be assertive | **Session 5**  45 minutes  Videocall  Child and parent | Fighting fears by facing fears |
| **Session 6**  45 minutes  Web/phone  (Child focus with parent support) | Preparing for the future | **Session 6**  45 minutes  Videocall  Child and parent | Preparing for the future |
