# Supplementary material for: ADAPTed Cognitive Behavioral Therapy for Pediatric Functional Abdominal Pain in Community-Based Pediatric Care: Mixed Methods Study
Source: JMIR Form Res. 2025 Aug 20;9:e67106. doi: 10.2196/67106 (PMC12367232; doi:10.2196/67106)
Supplement: Multimedia Appendix 3 [file formative-v9-e67106-s003.docx]

| ***Introduction*** | |
| --- | --- |
| *You and I are going to talk about your experience of participating in ADAPT. If you don’t mind, I would like to record our conversation so that I can listen carefully to what you tell me and not have to write it down while we are talking. It is only I and three of my research colleagues that will listen to the voice recording and once it has been typed up we will deleted the recording. Is it ok with you that I record our conversation?*  *You can choose which questions you would like to answer and if you would like to take a break or end the interview it can be done at any time you wish. Is it ok if I start recording now?* | |
| **Prior to treatment** | |
| 1. | Can you describe some thoughts you had about ADAPT prior to treatment? |
| 2. | How did you feel about starting treatment? Did you talk about it at home? What did you think you would learn? |
| **Experience of participation** | |
| 3. | What was your experience of participating in ADAPT? |
| 4. | First you came to the clinic for some sessions with the psychologist, and then you watched films and did assignments at home. What was your experience of this? |
| 5. | What was your experience of the sessions with the psychologist in clinic? Did you learn anything? If so, what did you learn?  If necessary, more specific: What did you think of the strategies you were taught (breathing and relaxation techniques, Calming Statements, Activity Pacing) |
| 6. | What did you think of the content in the modules that you worked with at home (Pleasant activities and how to solve problems, Detective Thinking, Stepladders)? Did you learn anything? If so, what did you learn?  If necessary, more specific: What did you think of the animated films? What did you think of the assignments? What did you think of the strategies that you were taught (Pleasant activities and how to solve problems, Detective Thinking, Stepladders)? |
| 7. | What did you think of the video calls with the psychologist? |
| 8. | How did you do the work at home? |
| 9. | Did you do the work on your own or did somebody help you? If so who? In which way did you receive help |
| 10. | Was there anything in the treatment that you thought was lacking or wished was different? If so what? |
| **After the treatment** | |
| 11. | Is there anything that is different now that you have have finished the treatment? If so what? How do you notice?  If necessary more specific: How is your abdominal pain and worry now, after treatment? What are your thoughts about that? |
| 12. | Is there anything else you would like to tell me? |
| *Thank you for participating and talking to me about your experience of ADAPT!* | |
