# Supplementary material for: ADAPTed Cognitive Behavioral Therapy for Pediatric Functional Abdominal Pain in Community-Based Pediatric Care: Mixed Methods Study
Source: JMIR Form Res. 2025 Aug 20;9:e67106. doi: 10.2196/67106 (PMC12367232; doi:10.2196/67106)
Supplement: Multimedia Appendix 4 [file formative-v9-e67106-s004.docx]

| 1. | Have you been absent from school due to abdominal pain in the last 4 weeks? (If yes) How many days? |
| --- | --- |
| 2. | Have you gone home from school due to abdominal pain in the last 4 weeks? (If yes) How many times? |
| 3. | Have you skipped leisure activities due to abdominal pain in the last 4 weeks? (If yes) How many times? |
| 4. | Are you worried about your abdominal pain?  (If yes) What about your pain worries you? |
| 5 | Are you worried about other things?  (If yes) What do you worry about? |
| 6 | Do you feel stressed?  (If yes) What makes you feel stressed? |
| **7** | Have you changed you diet due to your abdominal pain?  (If yes) What have you changed? |
| 8 | Have you tried anything that improves your abdominal pain? |
