# Supplementary material for: ADAPTed Cognitive Behavioral Therapy for Pediatric Functional Abdominal Pain in Community-Based Pediatric Care: Mixed Methods Study
Source: JMIR Form Res. 2025 Aug 20;9:e67106. doi: 10.2196/67106 (PMC12367232; doi:10.2196/67106)
Supplement: Multimedia Appendix 5 [file formative-v9-e67106-s005.docx]

| **Theme** | **Subtheme** | **Quotes** |
| --- | --- | --- |
| Starting from scratch | No experience of psychological treatment | “I hadn’t really been told much about it before”  “No one really knew what was going to happen”  “I’d never had psychological treatment before”  “I’d never tried real treatment before” |
|  | Uncertain expectations | “I think it was leaning more towards the positive”  “I don’t really think I thought about what I was going to do. I didn’t have a clue”  “[Learning sic] What to do to decrease the pain”  “I don’t know… that we were going to sit and talk… I hadn’t been told much about it”  “I didn’t think it was going to work, I wasn’t really into it”  “First when I came here I thought it was going to be a waste of time”.  “I thought it might help but I didn’t know how it would help”  “So it was more like thinking whether I’d get help or not” |
| Experiencing the treatment | Getting on with the content | “I thought the films were very informative and I thought it was good to repeat them at home.”  “And the films, I thought it was good to hear someone else’s thoughts, the little boy who talked about what it was like for him”.  “And if I couldn’t watch the films, I could read the texts”.  “It went well, I understood the assignments and that.”  “Sometimes it was tricky but mostly it wasn’t really hard.”  “…I didn’t really relate to the examples.”  “Make the videos shorter and more to the point”  “It was hard to think about what to write.” |
|  | Engaging with a therapist | “The first time it was a bit awkward, I hadn’t been there before but it got better and better each time”  “It worked well [coming to the clinic sic], I think”  “Then you got to speak properly in person like, so I thought that was good”  “Its nice meeting in person”  “The [strategies sic] she showed me are the ones I use now so it was good that I first got an idea of how you do it in real life…” |
|  | Managing the digital format | “Doing the work at home worked well too, it’s nothing I haven’t done before.”  “Then it was like a school assignment, you answered questions and that…”  “[the video calls sic]…worked well, because they weren’t too long, it was just right”  “It was hard to get the mic to work a few times”.  “You could sit by the computer straight after school”  “Pretty much a huge time saver”  “You didn’t have to spend time getting there”.  “It was just nicer, I don’t know how to explain why but it was just nicer having short sessions”.  “You didn’t need to take time to travel there…”   “Doing the work at home could be tedious and boring… but it helped so I thought it was quite good.” |
|  | Collaborating with a parent/carer | “I didn’t understand the pain gate theory until my mum explained it”  “Then I asked my mum and she gave me suggestions.”  “It was both mum and dad, sometimes both of them, sometimes only mum, sometimes only dad”  “I often sat on my own but sometimes I got help from mum”  “It was only if I was stuck on something, and writing, she used to help me with that.”  “… I usually want to do work on my own, it’s easier for me anyway.”  “She [mum sic] used to watch the films with me.” |
| Getting on with life | Using some of a range of strategies | “When I get a tummy pain I use the muscle strategies.”  “I use them all but more subconsciously, almost every night when I go to sleep I do relaxation and I do deep breathing very much without really thinking about it.”  “I liked the breathing exercises very much and I like the muscle relaxation exercise”.  “I don’t use it a lot but I say it to myself sometimes… I kinda think it in my head”  “I kinda pace myself when I eat so I don’t eat too fast”  “I don’t think I did that one a lot but I have done it a few times. I don’t think I tried it enough”  “I think I already did that.”  “Relaxation and deep breathing works”.  “You get to learn a lot but all of them don’t work for everything”  “The fantasy one, thinking about something else”  “From my perspective they didn’t really work but maybe they would have if I understood it better.” |
|  | Learning about pain and worry | “It’s tough when you have to learn about your problems…my brain tells me the opposite”  “…. controlling it better so I can get through the day anyway.”  “I learned to think positively, I wasn’t good at positive thoughts.”  “I have learnt that it’s not gonna change that I still need to go to the toilet but I can hold on and don’t have to rush”  “I thought I wouldn’t have any more pain if it had worked but now I know that it is about taking away the worry about having pain…  I’m not worried about the pain anymore”.  “So I kinda learnt that it can be stress that affects my pain.” |
|  | Doing things despite of symptoms | “I haven’t stayed home from school, so it has helped”  “I do go to school very very much more”  “Going to school even if I have a tummy ache”  “I do my regular things even if I have my tummy pain  “I have done stuff even if my tummy hurt”  “…most things were about avoiding activities and that but I already do things so it hasn’t changed a lot for me” |
